# Polyphasic study of phytopathogenic bacterial strains associated with deep bark canker of walnut in Serbia revealed a new species, *Brenneria izbisi* sp. nov

**DOI:** 10.3389/fpls.2022.1055186

**Published:** 2022-11-24

**Authors:** Katarina Gašić, Nevena Zlatković, Nemanja Kuzmanović

**Affiliations:** ^1^ Department of Plant Diseases, Institute for Plant Protection and Environment (IPPE), Belgrade, Serbia; ^2^ Julius Kühn Institute (JKI), Federal Research Centre for Cultivated Plants, Institute for Plant Protection in Horticulture and Urban Green, Braunschweig, Germany

**Keywords:** *Juglans regia*, bacterial disease, MLSA, ANI, primer design, whole-genome sequencing, *Brenneria*, *Pectobacteriaceae*

## Abstract

Serious outbreaks of walnut deep bark canker were observed on young walnut trees (*Juglans regia* L.) in two localities in the northern part of Serbia during 2020. From the symptomatic walnut tissues, two types of bacterial colonies were isolated, predominantly, light cream, circular and smooth colonies, as well as small, yellowish, mucoid and convex ones. PCR analysis and phenotypic assays suggested that the former group belongs to *Brenneria* spp., while the latter isolates were identified as *Xanthomonas arboricola* pv. *juglandis*. Within the *Brenneria* group, two strains were identified as *Brenneria nigrifluens*, while other 15 strains did not belong to any *Brenneria* species described so far. Therefore, we selected four representative strains of the unknown *Brenneria* sp. and subjected them to polyphasic analysis. As expected, in a phylogenetic tree based on partial 16S rDNA sequences, four novel strains grouped with other *Brenneria* representatives, and showed close phylogenetic relationship to *Brenneria salicis*. Furthermore, multilocus sequence analysis (MLSA) based on the partial sequences of *atpD, gyrB*, *infB* and *rpoB* housekeeping genes and core-genome phylogeny indicated that the studied strains form a novel and a clearly separate *Brenneria* lineage. Overall genome relatedness indices showed that they represent a new *Brenneria* species. The new species can be differentiated from the other *Brenneria* spp. infecting walnut and closely related *B. salicis* strains based on phenotypic characteristics, as well. Moreover, the pathogenicity tests on two-year-old walnut plants proved the ability of strains to cause necrosis and longitudinal black lesions and cracks on the trunk and branches of walnut trees. Overall, polyphasic characterization showed that the studied strains isolated from walnut with symptoms of deep bark canker represent a novel species of the genus *Brenneria* for which the name *Brenneria izbisi* sp. nov. is proposed. The type strain of *B. izbisi* is KBI 423^T^ (= CFBP 9035^T^ = LMG 32479^T^). To facilitate rapid identification of newly described species, a conventional PCR protocol and primers targeting the putative gene *hrpP*, were developed. Further study should reveal the potential role of each pathogen isolated from symptomatic walnut in disease development as well as possible interaction between them.

## Introduction

Global demand for tree nuts has increased considerably in recent years, leading to expansion of planted area of the almond, walnut, and hazels, as the three major nut crops. Walnut is considered as the most important nut crop worldwide, of which production is constantly increasing. According to FAOSTAT (2022) ^1^, the area of the walnut orchards reached 1.02 million ha, with total production 3.3 million tones in shell at global level in 2020. In Serbia, interest in English (Persian) walnut (*Juglans regia* L.) production has recently expanded due to the market and export potential. The total walnut planted area in 2017 was 3.307 ha, reaching 12.276 t of total yield, according to Statistical Office of the Republic of Serbia^2^. Commercial walnut plantations were mainly established with imported propagation material, mostly originating from Turkey, which is the fourth biggest producer after China, USA and Iran (FAOSTAT, 2022)^1^.

Walnut production can be compromised by different biotic factors, including plant pathogenic bacteria. *Xanthomonas arboricola* pv. *juglandis* (*Xaj*) (Vauterin et al., 1995) is the causal agent of walnut bacterial blight (WBB), the most important bacterial disease of walnut worldwide, including Serbia (Du Plessis and van der Westhuizen, 1995; Scortichini et al., 2001; Kałuzna et al., 2014; Ivanović et al., 2015; Giovanardi et al., 2016; Fu et al., 2018). The symptoms of WBB can be observed on all aboveground organs, including leaves, twigs, catkins and fruits. *Xaj* has been also associated with symptoms of brown apical necrosis (BAN) at the stigmatic end of the fruit (Belisario et al., 2002; Moragrega and Özaktan, 2010; Moragrega et al., 2011), and vertical oozing canker (VOC) developed in woody tissue (Hajri et al., 2010). Symptoms of VOC are characterized by development of vertical cankers on affected trunks and branches, with oozing exudates occurring mainly during summer (Hajri et al., 2010). Other pathogens affecting walnut trunks and branches belong to the genus *Brenneria*. *Brenneria nigrifluens* (*Bn*) (Wilson et al., 1957; Hauben et al., 1998) is the causal agent of shallow bark canker, while *Brenneria rubrifaciens* (*Br*) causes deep bark canker on walnut (Wilson et al., 1967). Both diseases show similarities in external canker symptoms and production of black exudate (sap wood) and were first occurred in the USA (Wilson et al, 1957; Wilson et al, 1967). In Europe, *Bn* was first recorded in Spain (López et al., 1994), followed by its occurrence in Italy (Saccardi et al., 1998; Morone et al., 1998), Serbia (Popovic et al., 2013), France (Ménard et al., 2004), Hungary (Végh et al., 2014), and recently in Turkey (Soylu et al., 2021). On the other hand, *Br* was reported, for the first time in Europe, on walnut trees in Spain imported from California (González et al., 2002).

So far, among phytopathogenic bacteria causing disease on walnut in Serbia, *Xaj* was detected and characterized as a causal agent of WBB (Gavrilović and Arsenijević, 1998; Ivanović et al., 2015). In 2013, Popovic et al. (2013) reported the presence of *Bn* in a 30-year-old walnut orchard in the Fruška Gora region. However, recently, unusual symptoms of bark canker were observed on four-year-old walnut trees (*Juglans regia* L.) cv. Chandler in a 1-ha orchard in northern part of Serbia, province of Vojvodina (Vrbas) in September 2020. Disease symptoms included dark brown to black, roundish, sunken lesions on the bark of trunks and lower branches. Affected bark developed longitudinal cracks that oozed a dark liquid, leaving glossy dark traces just below the wounds. Removing the bark revealed extensive discoloration of the tissue in longitudinal blackish lesions that extended deeper into the xylem. On some trees, progressive infection resulted in cankers that spread along the trunk, subsequently killing the tree. The incidence of the disease was approx. 70%, and 1% of the trees died in the time of sampling. Subsequently, similar symptoms of disease were observed on four-year-old walnut trees in another walnut orchard (Sivac, 8 ha), in the same region. Propagation material, two-year old walnut trees, for both orchards was imported. The primary objective of the present study was to identify and characterize bacteria associated with the new walnut disease in Serbia.

## Materials and methods

### Plant sampling and bacterial isolation

**Figure 1:**
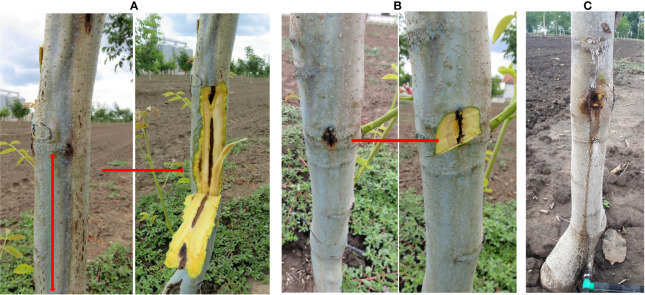
Symptoms of deep bark canker on walnut, before and after bark removal. **(A)** Dark brown to black, sunken longitudinal lesions under the bark of walnut; **(B)** Extensive discoloration of the tissue that extend deeper into the xylem; **(C)** Longitudinal oozing cracks. Natural infection.

**Figure 2:**
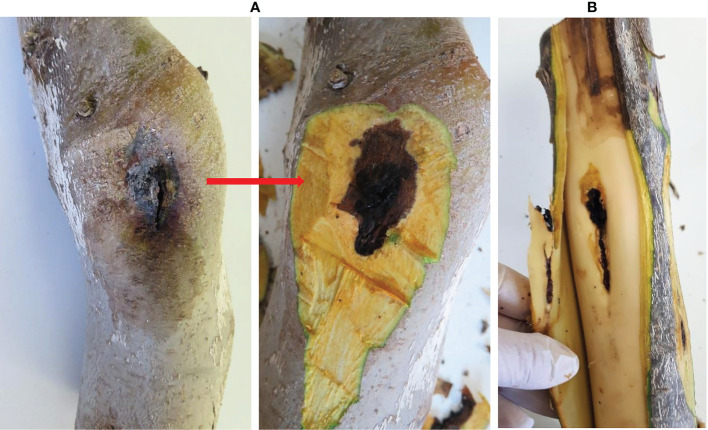
Isolation from the symptomatic walnut samples. **(A)** Dark, roundish, sunken lesions before and after removing the bark; **(B)** Necrotic lesions under the bark extended into the xylem tissue. Natural infection.

The samples that contained parts of diseased walnut branches and trunks showing symptoms of deep bark canker and longitudinal oozing cracks (**Figures 1**, **2**) were collected in orchards in the north part of Serbia in the late summer (September, 2020). Bacterial isolation was carried out from several sections of the symptomatic walnut tissue. Briefly, fresh fragments from border area between apparently healthy and diseased tissue under the bark and deeper in the region of the xylem, were macerated in 1 ml of sterile distilled water (SDW). The macerate was incubated at room temperature for 10 min and streaked onto King’s medium B (KB) (King et al., 1954), followed by incubation at 27°C. The sapwood samples found in small cracks were also used for isolation on KB medium. After 3 days, bacterial colonies were purified and maintained on KB medium for further testing. Long-term storage of bacterial isolates was achieved at -80°C in a mixture of nutrient broth medium and 30% glycerol (Schaad et al., 2001).

Sources of bacterial strains used within this study, including reference strains, are listed in **Table 1** and **Table S1**.

**Table 1:** Bacterial strains used in this study and results of PCR analysis.

						PCR analysis				
Strain	Species	Year of isolation	Origin	Host plant	Source^a^	F1/C3	BR1/BR3	Es1A/Es4B	Bi-hrpP-F/Bi-hrpP-R	XajF/XajR
KBI 422	*Brenneria izbisi*	2020	Vrbas, Serbia	*Juglans regia*	This study	–	–	+	+	NT
KBI 423^T^ (=LMG 32479^T^, =CFBP 9035^T^)	*Brenneria izbisi*	2020	Vrbas, Serbia	*Juglans regia*	This study	–	–	+	+	NT
KBI 424	*Brenneria izbisi*	2020	Vrbas, Serbia	*Juglans regia*	This study	–	–	+	+	NT
KBI 425	*Brenneria izbisi*	2020	Vrbas, Serbia	*Juglans regia*	This study	–	–	+	+	NT
KBI 426	*Brenneria izbisi*	2020	Vrbas, Serbia	*Juglans regia*	This study	–	–	+	+	NT
KBI 427	*Brenneria izbisi*	2020	Vrbas, Serbia	*Juglans regia*	This study	–	–	+	+	NT
KBI 428	*Brenneria izbisi*	2020	Vrbas, Serbia	*Juglans regia*	This study	–	–	+	+	NT
KBI 429	*Brenneria izbisi*	2020	Vrbas, Serbia	*Juglans regia*	This study	–	–	+	+	NT
KBI 430	*Brenneria izbisi*	2020	Vrbas, Serbia	*Juglans regia*	This study	–	–	+	+	NT
KBI 431	*Brenneria izbisi*	2020	Vrbas, Serbia	*Juglans regia*	This study	–	–	+	+	NT
KBI 432	*Brenneria izbisi*	2020	Vrbas, Serbia	*Juglans regia*	This study	–	–	+	+	NT
KBI 433	*Brenneria izbisi*	2020	Vrbas, Serbia	*Juglans regia*	This study	–	–	+	+	NT
KBI 434	*Brenneria izbisi*	2020	Vrbas, Serbia	*Juglans regia*	This study	–	–	+	+	NT
KBI 447	*Brenneria izbisi*	2020	Sivac, Serbia	*Juglans regia*	This study	–	–	+	+	NT
KBI 448	*Brenneria izbisi*	2020	Sivac, Serbia	*Juglans regia*	This study	–	–	+	+	NT
KBI 446	*Brenneria nigrifluens*	2020	Sivac, Serbia	*Juglans regia*	This study	+	–	–	–	NT
KBI 449	*Brenneria nigrifluens*	2020	Sivac, Serbia	*Juglans regia*	This study	+	–	–	–	NT
KBI 435- KBI 439	*Xanthomonas arboricola* pv. *juglandis*	2020	Vrbas, Serbia	*Juglans regia*	This study	NT	NT	NT	NT	+
KBI 440- KBI 445	*Xanthomonas arboricola* pv. *juglandis*	2020	Sivac, Serbia	*Juglans regia*	This study	NT	NT	NT	NT	+
KBI 024	*Brenneria nigrifluens*	2011	Serbia	*Juglans regia*	KBI	+	–	–	–	NT
LMG 2709^T^	*Brenneria rubrifaciens*	unknown	USA	*Juglans regia*	LMG	–	+	–	–	NT
LMG 2698^T^	*Brenneria salicis*	1957	UK	*Salix alba*	LMG	–	–	+	–	NT

a KBI, Collection of Bacteria, Institute for Plant Protection and Environment, Belgrade, Serbia; LMG, Bacteria Collection, Ghent University, Belgium. -, no amplification product observed on agarose gel; +, production of the specific amplification fragment from genomic DNA; NT- non tested.

### Physiological and biochemical tests

In order to identify and characterize isolates, various physiological and biochemical tests were performed. They were tested for Gram reaction using 3% KOH, catalase activity, fluorescence on KB, oxidative–fermentative (O/F) test, oxidase activity, levan production, pectinolytic activity on potato slices and production of a hypersensitive reaction (HR) on tobacco (*Nicotiana tabacum* cv. ‘Samsun’) and geranium (*Pelargonium* x *hortorum*) leaves (Lelliott and Stead, 1987). The isolates were grown on different media such as KB, Yeast extract - Dextrose-Calcium carbonate agar (YDC), Yeast-Peptone-Glucose Agar (YPGA), Nutrient Agar supplemented with 5% sucrose w/v (NSA) (Schaad et al., 2001) Additional biochemical and physiological analyses of seven walnut isolates (KBI 423^T^, KBI 424, KBI 429, KBI 430, KBI 447, KBI 446 and KBI 449), including two reference strains LMG 2709^T^ of *Brenneria salicis* (*Bs*) and LMG 2698^T^ of *Br*, were conducted using the API 20E, API 20NE, and API 50CH systems (bioMérieux). For API 20E and API 20NE, the pure bacterial cultures grown on KB medium for 24 h were suspended in 0.85% NaCl solution. Concentration of bacterial suspensions was adjusted to ~10^8^ CFU/ml photometrically (OD_600_ = 0.3). Further, the test strips were handled by following manufacturer instructions. For API 50CH, the bacteria were suspended in API 50 CHB/E medium (bioMérieux) in the same concentration. API strips were incubated at 27°C for 48 h (Biosca and López, 2012). Obtained results were compared with data available in the literature (Brady et al., 2012; Brady et al., 2014). All API tests were conducted in three replicates.

### Pathogenicity assay

Pathogenicity of the isolates was assessed by inoculating two-year-old walnut plants cv. Chandler grown in pots, by method of Moretti et al. (2007), with slight modifications. Six *Brenneria* isolates (KBI 423^T^, KBI 424, KBI 429, KBI 446, KBI 447 and KBI 449) and one isolate of *Xaj* (KBI 435) were selected for testing. The plants were inoculated by infiltration of 20 µl of bacterial suspension (~10^8^ CFU/ml, OD_600_ = 0.3) into 2-cm-long vertical stem wounds made aseptically by a scalpel. Two plants with three wounds (inoculation points), per each strain were inoculated. SDW was used as a negative control. Inoculated tissue was covered by wet cotton pad and wrapped with a Parafilm tape for four days to prevent drying out. One month post-inoculation, new, young saplings (shoots) were developed on the test plants. They were also inoculated by above described method with a slight modification – 1 cm longitudinal wound was made by scalpel and there were two inoculation sites per plant. Test plants were maintained in the greenhouse for 2 months. Afterwards, the pots were moved outside and observed for the next 14 months. The appearance of external and internal symptoms was recorded two and 14 months post-inoculation. Re-isolation of bacteria from inoculated plants (two and 14 months post-inoculation) was performed on KB medium and their identity was confirmed by PCR assay.

Additionally, pathogenicity of the representative isolates (KBI 423^T^, KBI 446 and KBI 435) was tested on immature walnut fruits prior to crust hardening. After surface sterilization with 70% alcohol, the fruits were injured on three sites by sterile tip and inoculated with 30 µl of tested bacterial suspension (~10^8^ CFU/ml). The test was performed in three replications. The reference strain of *Bn* KBI 024 (Popovic et al., 2013), type strains LMG 2709^T^ of *Br* and LMG 2698^T^ of *Bs*, were used as controls. SDW was used as a negative control. The fruits were placed in a sterile, humid, plastic chamber and incubated at 27°C for 15 days in dark conditions. Bacteria were re-isolated on KB and their identity was confirmed by PCR assay.

### DNA extraction

Total genomic DNA was extracted from pure bacterial cultures using the cetyl trimethyl ammonium bromide (CTAB) protocol, according to the instructions described by Angelini et al. (2001), with slight modifications. Bacterial suspensions (~10^9^ CFU/ml) were prepared in 500 µl 10 mM phosphate saline buffer (Na_2_HPO_4_ × 12 H_2_O 2.7 g/l, NaH_2_PO_4_ × 2 H_2_O 0.4 g/l). Afterwards, 500 µl extraction buffer (3% CTAB, 1M Tris-HCL pH 8.0, 1.4 M NaCl, 20 mM EDTA, 3% PVP) was added in the equal volume of bacterial suspensions and incubated at 65°C for 20 min. The following steps were as described in original protocol.

For whole-genome sequencing, DNA of isolates KBI 423^T^ and KBI 447 was extracted using the DNeasy Plant Mini Kit (Qiagen, Hilden, Germany). The quality of extracted DNA was checked by gel electrophoresis on 0.8% agarose gel and then stored at −20°C for further analysis.

### PCR analyses

Based on host plant, disease symptoms and colony morphology, we assumed that causal agents of infection might belong to genera *Brenneria* and/or *Xanthomonas*. Therefore, three PCR reactions were performed by using: *Bn-*specific primers F1/C3 (Loreti et al., 2008); primer pair BR1/BR3 specific for *Br* (McClean et al., 2008) and XajF/XajR primers specific for *Xaj* (Gironde et al., 2009). Additionaly, we applied a PCR with primers Es1A/Es4B, specific for *Bs* (Hauben et al., 1998). Amplified PCR products (5 µL) were separated by gel electrophoresis in 1.5% agarose gel in 0.5 × Tris-Borate-EDTA (TBE) buffer stained with 2% (v/v) Midori Green (MIDORI Green Advance, NIPPON Genetics EUROPE) and visualized by a digital imaging camera (Vilber Lourmat, France). All primers sequences used in this study are shown in **Table S2**.

### Phylogenetic analysis of 16S rRNA and housekeeping genes

The 16S rRNA and housekeeping gene fragments were amplified and sequenced (Macrogene Europe, The Netherlands) for several representative strains. The partial sequence of 16S rDNA of strains KBI 423^T^, KBI 424, KBI 428 and KBI 429 was amplified using universal pair of primers fD1 and rP2 (Weisburg et al., 1991). Four representative *Brenneria* strains (KBI 423^T^, KBI 429, KBI 430 and KBI 447) belonging to the unknown species were selected for multilocus sequence analysis (MLSA). PCR amplification and partial sequencing of four housekeeping genes, *atpD, gyrB, infB*, and *rpoB*, was conducted according to the protocol of Brady et al. (2008).

Chromatograms were visualized using FinchTV 1.4.0 software and sequences were processed using the MEGA 7 package (Kumar et al., 2016). In order to align the sequences, CLUSTAL W algorithm (Higgins et al., 1996) integrated into MEGA 7 software (Kumar et al., 2016) was used. For comparative analysis of the obtained sequences with the sequences deposited in the NIH GenBank, the BLASTn program was used (Altschul et al., 1997). The maximum likelihood (ML) phylogenies based on 16S rDNA and concatenated sequences of housekeeping genes were inferred using IQ-TREE 1.6.12 (Nguyen et al., 2015) software available through the IQ-TREE web server^3^ (Trifinopoulos et al., 2016). A model selection was conducted using IQ-TREE ModelFinder (Kalyaanamoorthy et al., 2017). The best-fit DNA substitution models HKY+F+I+G4 (16S rDNA) and TIM2+F+I+G4 (housekeeping genes, concatenated dataset) were selected based on Bayesian Information Criterion (BIC). Branch support was assessed by ultrafast bootstrap analysis (UFBoot) using 1000 replicates (Hoang et al., 2018). The tree was visualized using FigTree, v1.4.4^4^ (Rambaut, 2018).

### Strains differentiation by rep-PCR

Genetic relatedness among the strains was evaluated by rep-PCR fingerprinting. Seventeen *Brenneria* spp. isolates recovered within this study were amplified in three PCR reactions by BOXA1R, REP1R-I/REP2-I and ERIC1R/ERIC2 primers (Versalovic et al., 1991; Versalovic et al., 1994). Amplified fragments were separated by gel electrophoresis in 1.5% agarose gel in 0.5 × TBE buffer and visualized as described above.

### Whole-genome sequencing, assembly and annotation

Whole-genome sequencing was conducted for two *Brenneria* isolates, KBI 423^T^ and KBI 447. The library preparation, sequencing and initial processing of reads was performed by Novogene, Cambridge, United Kingdom. The genomic DNA was randomly sheared into short fragments (~350bp). DNA libraries were prepared with NEBNext Ultra II DNA Library Prep Kit for Illumina (New England Biolabs). Sequencing was performed on Illumina NovaSeq PE150 platform. A total of 2×8,784,885 and 2×8,152,797 paired-end reads were generated for strains KBI 423^T^ and KBI 447, respectively. Furthermore, adapter trimming and quality filtering of raw reads were conducted with Cutadapt ver. 3.7 (Martin, 2011). *De novo* sequence assembly was performed using SPAdes 3.15.3 (with options -k 21,33,55,77 and –isolate) (Prjibelski et al., 2020). Short (<200 bp) and low-coverage (<3-fold) contigs were discarded. Furthermore, the Illumina reads were mapped to the remaining contigs with BWA-MEM (Galaxy Version 0.7.17.2) (Li, 2013), to manually curate assemblies, involving correction of sequence errors and mis-assemblies. The genome sequences were annotated using Prokka (Galaxy Version 1.14.6+galaxy1) (Seemann, 2014) and NCBI Prokaryotic Genomes Annotation Pipeline (PGAP) (Tatusova et al., 2016).

### Core-genome phylogenetic analysis

Core-genome phylogeny was inferred using the GET_HOMOLOGUES Version 11042019 (Contreras-Moreira and Vinuesa, 2013) and GET_PHYLOMARKERS Version 2.2.8_18Nov2018 (Vinuesa et al., 2018) software packages as described before (Kuzmanović et al., 2022). The dataset contained two strains studied (KBI 423^T^ and KBI 447), as well as reference strains of *Brenneria* (14 strains), *Dickeya* (3 strains), *Lonsdalea* (3 strains) and *Pectobacterium* (3 strains). As an outgroup, we used members of the family *Erwiniaceae*, including *Erwinia* spp. (4 strains) and *Pantoea* spp. (4 strains) (**Table S3**).

### Overall genome relatedness indices

The species delineation was assessed by different overall genome relatedness indices (OGRIs), including the average nucleotide identity (ANI) (Goris et al., 2007) and digital DNA-DNA hybridization (dDDH) (Meier-Kolthoff et al., 2013). The ANI calculations were performed using PyANI program Version 0.2.11, with scripts employing BLAST+ (ANIb) and MUMmer (ANIm) algorithm to align the input sequences^5^ (Pritchard et al., 2016), and OrthoANIu Version 1.2 (calculates orthologous ANI using USEARCH algorithm) (Yoon et al., 2017). The dDDH values were computed by the Genome-to-Genome Distance Calculator (GGDC 2.1)^6^ using the recommended BLAST+ alignment and formula 2 (identities/HSP length) (Meier-Kolthoff et al., 2013).

### Development of the specific PCR assay

For molecular detection, identification and differentiation of novel *Brenneria* strains characterized in this study, we developed a specific PCR assay. Specific primers were designed based on the sequence of the putative gene *hrpP* (locus_tag=“NC856_01210” in assembly referring to strain KBI 423^T^) coding for type III secretion system HrpP C-terminal domain-containing protein. Candidate primers were identified at nucleotide positions 90–114 and 407–429 and were designated Bi-hrpP-F (5′-TGATAGCTTTTGGGAAGAGTTCGCT-3′) and Bi-hrpP-R (5′-ACAGGATTCACGCCGATCTTTCA-3′), respectively. The PrimerQuest tool by Integrated DNA technologies (IDT) was used to design primers (Integrated DNA Technologies, Coralville, IA). The size of the amplification product is 340 bp. Specificity of the primer set was tested with 46 strains (**Table S1**) and additionally checked *in silico* by performing NCBI Primer-BLAST against the non-redundant (nr) and “Refseq representative genomes” databases^7^.

The PCR amplifications were performed in a 15 μl mixture containing: 1 × Color OptiTaq PCR Master Mix (EURx, Gdańsk, Poland) (1.25 U OptiTaq DNA Polymerase; 1× Reaction Buffer with 1.5 mM MgCl_2_; 0.2 mM of each dNTP), 0.5 μM of each primer and 1.5 µl template DNA. The thermal profile was as follows: initial denaturation at 95˚C for 2 min, 35 cycles of denaturation at 95˚C for 30 s, annealing at 60˚C for 30 s, elongation at 72˚C for 30 s, and final extension at 72˚C for 5 min. Reaction were performed in a Thermal Cycler 2720 (Applied Biosystems, Foster City, CA, USA).

### Accession numbers

The GenBank accession numbers for the partial 16S rRNA gene sequences of the Brenneria strains KBI 423T, KBI 424, KBI 428 and KBI 429 are MW485945 to MW485948, respectively. Generated (**Table S3**) partial sequences of housekeeping genes for Brenneria strains KBI 447, KBI 430, KBI 429, KBI 423T, were deposited in the NIH GenBank database under accession numbers ON228202 to ON228205 for atpD gene, ON638957 to ON638960 for infB, ON638961 to ON638964 for gyrB and ON638965 to ON638968 for rpoB, respectively (**Table S3**).

The Whole Genome Shotgun projects have been deposited at DDBJ/ENA/GenBankunder the accessions JAMPJT000000000 (KBI 447) and JAMPJU000000000 (KBI 423T); within the BioProject PRJNA832546. The versions described in this paper are the first versions, JAMPJT010000000 and JAMPJU010000000. The raw sequencing reads were deposited in the Sequence Read Archive (SRA) under the same BioProject PRJNA832546.

## Results

### Sampling, isolation and phenotypic characterization of bacterial strains

Bacterial strains were isolated from the symptomatic walnut samples collected in two localities in the northern part of Serbia. After 2 to 3 days of incubation at 27°C on KB medium, two major types of colonies were observed. Light cream, circular and smooth colonies with entire margins were predominant on the KB plates. The second group consisted of small, yellowish, circular and convex colonies resembling *Xanthomonas* species. The former strains were Gram- and oxidase negative, facultatively anaerobic, levan positive, nonfluorescent, and did not produce bright-red pigment on YDC and YPGA medium. Seventeen tested isolates showed phenotypic characteristics as described for type *Brenneria* spp. (**Table 1**) (Brady et al., 2012). In particular, they showed no pectinolytic activity on potato slices. They did not induce hypersensitive reaction on tobacco leaves but induced HR reaction on geranium 24 h after inoculation. Strains KBI 446 and KBI 449 did not induce HR response in any test plant. No symptoms were observed on tissue inoculated with SDW.

Seven representative strains were selected for further analysis by API 20E, API 20NE, and API 50CH tests and compared with strain LMG 2698^T^ of *Bs*, LMG 2709^T^ of *Br*, as well as literature data for *B. goodwinii*, *B. alni*, *B. rosae* subsp. *rosae* and *Lonsdalea quercina* (Brady et al., 2012; Brady et al., 2014). Five representative strains (KBI 423^T^, KBI 424, KBI 429, KBI 430 and KBI 447) showed phenotypic traits as follow: positive reactions for acid production from L-arabinose, D-galactose, inositol, melibiose, raffinose, potassium gluconate, glycerol, and negative reactions from gentiobiose, D-sorbitol, trehalose, turanose, amygdalin and D-xylose (**Table 2**). The obtained results were compared with features already determined for other *Brenneria* species (Brady et al., 2012), and we found that these strains showed unique characteristics as they did not fit into any already existing profile. Two remaining strains (KBI 446 and KBI 449) produced acid from L-arabinose, gentiobiose, inositol, melibiose, raffinose, D-sorbitol, trehalose, glycerol and D-xylose and showed negative reaction for amygdalin, indol, turanose, D-galactose, and potassium gluconate. These two strains had identical features as *Bn* previously described (Brady et al., 2012) (**Table 2**).

**Table 2:** Differential phenotypic characteristics for *B. izbisi* and other *Brenneria* and *Lonsdalea* species.

Characteristic	Species							
	*B. izbisi*sp. nov.	*B. salicis*	*B. rubrifaciens*	*B. roseae* ssp. *roseae*	*B. nigrifluens*	*B. goodwinii*	*B. alni*	*L. quercina*
	KBI 423^T^, 424, 429, 430, 447	LMG 2698^T^	LMG 2709^T^	LMG 27714^T^	KBI 446, 449	FRB 141^T^	NCPPB 3934^T^	ATCC 29281^T^
L-Arabinose	+	–	+	+	+	+	+	–
Amygdalin	–	–	–	–	–	+	+	–
D-Galactose	+	–	–	+	–	+	+	d
Gentiobiose	–	–	–	–	+	+	–	–
Inositol	+	+*	–	+	+	+	–	–
Melibiose	+	+*	–	–	+	+	–	–
Potassium gluconate	+	+	–	+	–	d	–	d
Raffinose	+	+	–	–	+	+	–	–
D-Sorbitol	–	–	–	+	+	+	–	–
Trehalose	–	–	–	–	+	+	+	d
Turanose	–	+*	–	–	–	+	+	+
D-Xylose	–	–	–	+	+	d	+	–
Glycerol	+	–	–	+	+	+	+	–

All data were obtained using API 50CH test kit (bioMérieux) under same conditions. Tested strains of *B. izbisi* sp. nov and *B. nigrifluens* were isolated in this sudy, while *B. rubrifaciens* and *B. salicis* reference strains were used as control strains. Data for reference strains *B. goodwinii* were taken from Denman et al., 2012, for *B. alni* and *Lonsdalea quercina* were taken from Brady et al., 2012, and for *B. rosae* subsp. *rosae* from Brady et al., 2014. +, positive; -, negative; d- 11-89% strains positive in 1-4 days; *- differ from literature data.

Eleven *Xanthomonas*-like isolates belonging to the second group were aerobic, Gram- and oxidase negative, catalase positive, forming yellow, round, mucoid and convex colonies on YPGA medium after 3 days of incubation at 27°C. These strains induced HR reaction on both, tobacco and geranium leaves.

### Pathogenic properties of strains

In pathogenicity assay, extensive stem tissue necrosis developed around the point of inoculation of walnut plants two months after inoculation with the novel *Brenneria* strains KBI 423^T^, KBI 424, KBI 429 and KBI 447. Necrotic lesions were also observed in the inner bark tissue (**Figure S1A**). Lesions extended from the site of inoculation and small longitudinal cracks in the bark were formed. After bark removal, brown necrotic streaks extending deeper into the xylem were visible. After 14 months, the necrotic tissue under the bark of inoculated stems extended to 8 cm in length (**Figure S1B**). However, external cankers like those observed under natural conditions as well as oozing of a dark liquid from the wounds were not observed. Additionally, severe symptoms of deep tissue necrosis around the point of inoculation were observed on young branches three weeks after inoculation (**Figure S1C**). After removing the surface tissue, lesions appeared as longitudinal blackish streaks that extended deeper into inner tissue. Symptoms of internal necrosis were also observed in the plants inoculated with strains KBI 446 and KBI 449 of *Bn*. Strain KBI 435 of *Xaj* caused limited necrosis of stem and shoots tissue at point of inoculation. From all inoculated plants, bacteria were re-isolated on KB medium from the lesions on the stem and branches, respectively two and 14 months after inoculation. Identity of the strains was confirmed by specific PCR tests. Symptoms of necrosis did not develop on control plants, and we did not isolate any bacteria from the inner tissue.

In addition, characteristic symptoms of necrosis and sunken black lesions around the point of inoculation and deeper in the inner tissue were observed on young walnut fruits 14 days after inoculation with strains KBI 423^T^, KBI 446 and KBI 435. Three weeks after inoculation, bacteria were re-isolated on KB and their identity was confirmed by specific PCR assays, described below. No symptoms developed on control fruits inoculated with *Bs* and SDW.

### PCR identification

Different PCR assays were applied in order to identify isolates. Seventeen isolates of *Brenneria* sp. were PCR tested by using primers specific to *Bn*, *Br* and *Bs*. In PCR assay using primers Es1A/Es4B specific for *Bs*, a 553 bp DNA fragment was amplified in fifteen isolates (**Table 1**). PCR reaction with F1/C3 primers yielded specific 250 bp product in two remaining isolates (KBI 446 and KBI 449), indicating their affiliation to *Bn* species. Primers specific for *Br* did not amplify expected 409 bp fragment in any tested isolate. Identity of yellowish, small, circular colonies as *Xaj* was confirmed by using XajF/XajR primers amplifying specific 216 bp fragment (**Table 1**).

### Strains differentiation by rep-PCR

The obtained genetic PCR profiles showed that the 15 novel *Brenneria* isolates represent a homogenous group, by using BOXА1R, ERIC1R/ERIC2 and REP1R-I/REP2-I primers. The isolates formed a unique fingerprint profile that differs from the profiles of *Bn, Br* and *Bs* used in the analysis (data not shown). Moreover, two *Bn* isolates showed an identical profile to the *Bn* reference strain KBI 024.

### Phylogenetic analysis of 16S rRNA and housekeeping genes

**Figure 3:**
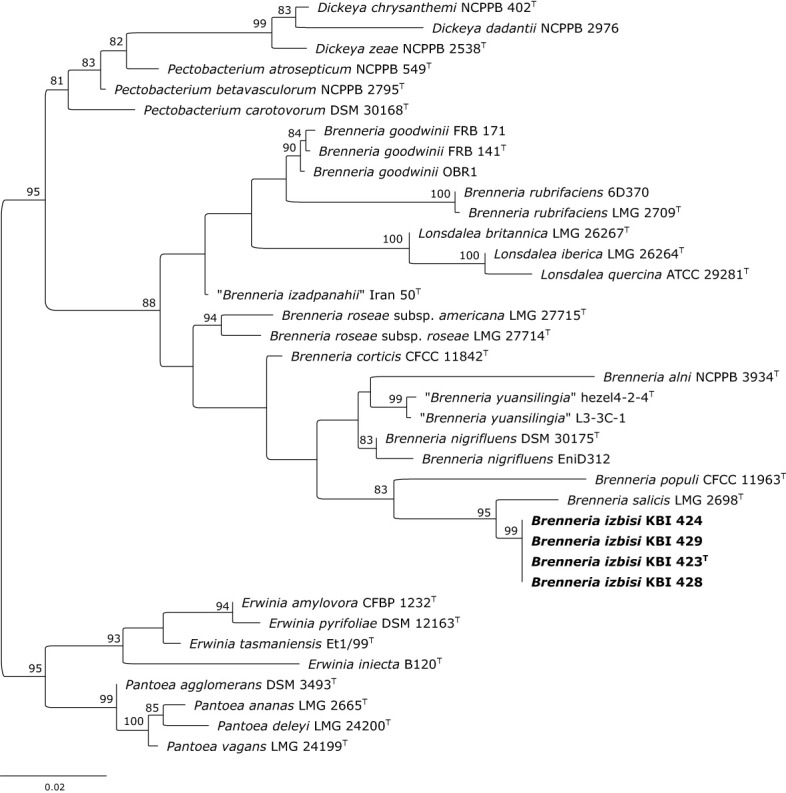
Maximum likelihood phylogenetic tree based on 16S rRNA gene sequence. Four representative *B. izbisi* strains isolated in this study are marked in bold. The best fitting model HKY+F+I+G4 was applied for tree construction. Ultrafast bootstrap support values (>70) are indicated at the nodes. Reference *Erwinia* spp. and *Pantoea* spp. were used as an outgroup to root the tree.

The partial 16S rRNA gene sequences of the novel *Brenneria* strains were identical, and showed highest nucleotide identity (98.42%) to the sequence of *Bs* type strain LMG 2698^T^ (Acc. No. AJ233419). Indeed, phylogenetic analysis based on 16S rDNA sequences also indicated that strains KBI 423^T^, KBI 424, KBI 428 and KBI 429 were most closely related to *Bs* (**Figure 3**), although they formed a separate phylogenetic lineage within the genus *Brenneria*. Moreover, 16S rDNA phylogeny indicated that the genus *Brenneria* is polyphyletic, as it was intertwined with *Lonsdalea* spp. (**Figure 3**).

**Figure 4:**
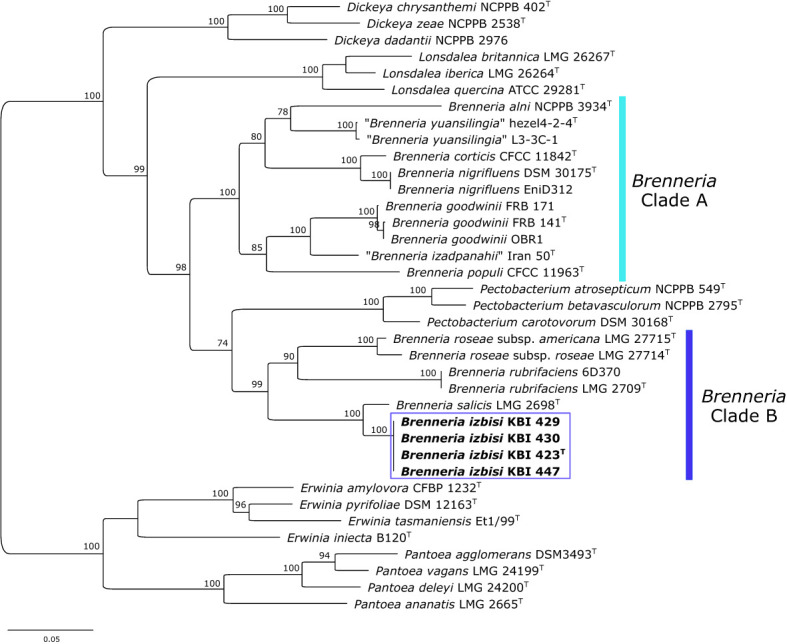
Maximum likelihood phylogenetic tree based on concatenated housekeeping genes *atpD, gyrB*, *infB* and *rpoB, (*2480 bp) sequences. The tree indicates the phylogenetic position of 4 representative *B. izbisi* strains (marked in bold and boxed). The best fitting model TIM2+F+I+G4 was applied for tree construction. Ultrafast bootstrap support values (>70) are indicated at the nodes. Reference *Erwinia* spp. and *Pantoea* spp. were used as an outgroup to root the tree.

Partial sequences of *atpD, gyrB*, *infB* and *rpoB* genes were obtained for four novel *Brenneria* isolates (KBI 423^T^, KBI 429, KBI 430 and KBI 447). Strains studied exhibited identical sequences for each of the four housekeeping genes. Phylogenetic trees were generated based on the sequence of each gene (data not shown), as well as on concatenated datasets of four housekeeping genes (**Figure 4**). In all phylogenetic trees, novel *Brenneria* strains formed a separate clade with 100% bootstrap support, with *Bs* located on an adjacent branch. In the phylogenetic tree generated from concatenated dataset, they clustered within the *Brenneria* clade B defined previously by Bakhshi Ganje et al. (2021) (**Figure 4**). Similarly as for 16S rRNA gene phylogeny, polyphyly of the genus *Brenneria* was evident in the MLSA tree. In particular, *Brenneria* clades A and B were separated by *Pectobacterium* strains included in the analysis (**Figure 4**).

### Whole-genome sequences

Draft genome sequences of strains KBI 423^T^ and KBI 447 were obtained in this study (**Table 3**). The *de novo* assembly resulted in 66 (KBI 423^T^) and 62 (KBI 447) contigs. The genome coverage was 638- (KBI 423^T^) and 592-fold (KBI 447). The draft genome sequence of strain KBI 423^T^ consisted of 3,896,770 bp, with an average G+C content of 51.76%, while that of strain KBI 447 consisted of 3,897,516 bp, with an average G+C content of 51.76%. Other general features of draft genome sequences are sumarized in **Table 3**.

**Table 3:** General features of genome sequences obtained in this study.

	Strains	
	*Brenneria izbisi* KBI 423^T^	*Brenneria izbisi* KBI 447
Contigs (N)	66	62
N50 (Kb)	279	279
Size (Mb)	3.9	3.9
GC Content (%)	51.8	51.76
Genes^a^	3,531	3,530
CDSs^a^	3,458	3,457
Accession number	JAMPJU000000000	JAMPJT000000000

a Numbers based on Prokka annotation.

### Core-genome phylogeny and overall genome relatedness indexes

**Figure 5:**
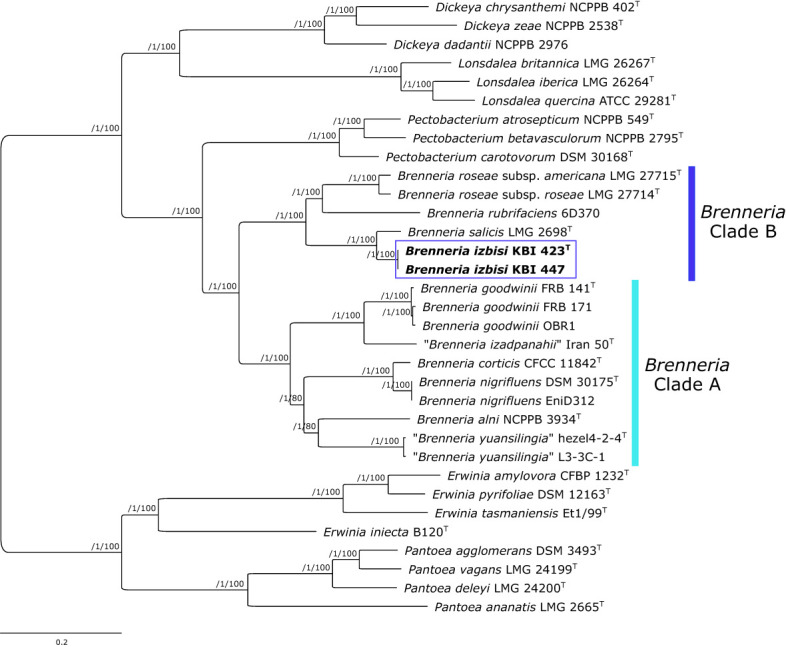
Maximum likelihood core-genome phylogenetic tree showing the evolutionary relationships between *Brenneria izbisi* (marked in bold and boxed) and related *Brenneria* spp. Reference *Dickeya*, *Lonsdalea* and *Pectobacterium* strains were also included in the analysis. Reference *Erwinia* spp. and *Pantoea* spp. were used as an outgroup to root the tree. The phylogeny was estimated under the GTR+F+ASC+R4 substitution model from the supermatrix obtained by concatenation of 663 top gene markers. The approximate Bayesian posterior probability values are shown at nodes. The scale bar represents the number of expected substitutions per site. DDBJ/ENA/GenBank whole-genome accession numbers are shown in **Table S3**. *Brenneria* clades A and B defined by Bakhshi Ganje et al. (2021) are indicated on the tree.

A core-genome phylogenetic tree was reconstructed from the supermatrix obtained by concatenation of 663 top gene markers. Strains KBI 423^T^ and KBI 447 formed a separate cluster within the genus *Brenneria*, within the clade B defined previously by Bakhshi Ganje et al. (2021) (**Figure 5**). As indicated by 16S rRNA and housekeeping gene phylogenetic analyses, core-genome phylogeny confirmed that their closest relative was *Bs* strain LMG 2698^T^ (**Figure 5**). Core-genome phylogeny indicated monophyly of the genus *Brenneria*, with two well-separated clades A and B.

OGRIs computed (**Table S4**) revealed that KBI 423^T^ and KBI 447 represent a new *Brenneria* species, closely related to *Bs*. In particular, they shared 94.48% (ANIb), 94.68% (ANIm), 94.51% (orthoANIu) and 57.8% (dDDH) overall genome relatedness with the type strain of *Bs* (LMG 2698^T^), which was below the proposed thresholds for species delineation (95–96% for ANI, and 70% for DDH) (**Table S4**). Obtained OGRIs were relatively low when KBI 423^T^ and KBI 447 were compared with other *Brenneria* spp. (<87% for ANI, <31 for dDDH). A description of the novel *Brenneria* species, for which the name *Brenneria izbisi* sp. nov. is proposed, is given below.

### Specific PCR assay for detection and identification of *Brenneria izbisi* sp. nov.

Genomic DNA of *B. izbisi* sp. nov. was amplified by using a conventional PCR assay with specific primers designed in this study. Using primers Bi-hrpP-F/Bi-hrpP-R, all tested strains of this species yielded the expected 340 bp amplicon. Contrarily, there was no amplification of DNA from the other 31 strains belonging to different species with this primer set (**Table S1**). Moreover, Primer-BLAST analysis against the nr database confirmed that the specificity of primers Bi-hrpP-F/Bi-hrpP-R is restricted to *B. izbisi*.

## Discussion

During a 2020, serious outbreak of deep bark canker was observed on young walnut trees in several orchards in Serbia. The symptoms included the development of deep longitudinal cracks in trunks and lower branches that oozed a dark liquid, leaving glossy dark traces just below the wounds. High incidence of disease was recorded in the newly established orchards suggesting that the pathogen was most likely disseminated by planting material. From the symptomatic walnut trees, strains belonging to three phytopathogenic bacterial species were isolated: *Bn*, *Xaj* and *Brenneria* strains that we described as a novel bacterial species named *B. izbisi* (see the protologue below).

Characterization of a newly isolated *Brenneria* species (*B. izbisi*) was the primary objective of this study. Among 15 strains showing identical phenotypic characteristic and rep-PCR fingerprints, we selected four representatives for a more comprehensive characterization. Partial 16S rDNA sequences of these four strains were identical and showed the highest nucleotide identity (98.42%) to the type strain of *Bs* (LMG 2698^T^). A nucleotide identity value for 16S rRNA gene sequences of 98.65% was proposed as the threshold for differentiating bacterial species (Kim et al., 2014). Distinctiveness of the four strains studied is also reflected in the 16S rRNA gene phylogenetic tree (**Figure 3**), where all four strains form a separate cluster with a strong bootstrap support, although a close phylogenetic relationship to *Bs* LMG 2698^T^ was evident.

An MLSA scheme based on partial gene sequencing of *atpD, gyrB*, *infB* and *rpoB* was proven to be useful for evaluation of the phylogenetic position of species belonging to the genus *Brenneria* within the *Enterobacteriaceae* (Brady et al., 2012). The present study revealed that *B. izbisi* strains had identical sequences for all analyzed genes. Based on the concatenated sequences of the four housekeeping genes (**Figure 4**), all four strains form a cluster with 100% bootstrap support, clearly separated from the closest phylogenetic neighbor *Bs*.

Although 16S rRNA gene and MLSA phylogenies indicated that *B. izbisi* strains belong to the genus *Brenneria*, this genus was polyphyletic in these phylogenetic trees. Polyphyly of the genus *Brenneria* in 16S rRNA gene phylogenetic tree has been observed previously (Brady and Coutinho, 2021). Therefore, we performed phylogenomic analysis based on a large number of non-recombinant core marker genes derived from whole-genome sequence data, in order to generate a more robust phylogenetic tree. In a resulting core-genome phylogenetic tree based on 663 gene markers, the genus *Brenneria* was monophyletic, with two well-separated clades A and B defined previously by Bakhshi Ganje et al. (2021). The separate phylogenetic position of *B. izbisi* was further supported by core-genome phylogeny. Lastly, the novel species could be unequivocally delineated from closely related species using different OGRIs, including ANI and dDDH.

Biochemical and physiological tests revealed that all strains possess basic characteristics of *Brenneria* genus. However, there are differential features that can be used to distinguish *B. izbisi* from other *Brenneria* species as shown in **Table 2**.

The specific focus of this study was to resolve the etiology of deep bark canker of walnut in Serbia and to assess the involvement of *B. izbisi* in infection and disease development. Currently, there are eight *Brenneria* species with validly published names (*B. salicis, B. alni, B. goodwinii, B. roseae, B. corticis, B. populi, B. nigrifluens* and *B. rubrifaciens*)^8^, including four subspecies (*Brenneria populi* subsp. *populi*, *Brenneria populi* subsp. *brevivirga*, *Brenneria roseae* subsp. *roseae* and *Brenneria roseae* subsp. *americana*) (Brady and Coutinho, 2021), as well as two species with not validly published names (“*Brenneria izadpanahii*” and “*Brenneria yuansilingia*”) (Bakhshi Ganje et al., 2021; Sun et al., 2021). Upon submission of this manuscript, Kile et al. (2022) described a new *Brenneria* species, *Brenneria tiliae* isolated from symptomatic *Tilia × moltkei* and *Tilia × europaea* trees in the UK. However, this species was distantly related to *B. izbisi* (ANIb < 81%). Its closest relative was *B. corticis* and unlike *B. izbisi*, *B. tiliae* clustered with strains of *Brenneria* clade A (Bakhshi Ganje et al., 2021; Kile et al., 2022). Overall, all *Brenneria* species described so far are associated with symptoms of bark canker and stem bleeding of the woody plants such as trees of alder, willow, poplar or walnut (Brady and Coutinho, 2021). Two of these species, *Bn* and *Br*, cause shallow and deep bark canker, respectively. They represent a serious threat to walnut production by the weakening of trees and consequent reduction in the number of nuts and in timber production, as well as reduced yield. Recently, Allahveripour et al. reported that *B. roseae* subsp. *rosae* and *Gibbsiella quercinecans* were associated to the shallow bark canker of walnut tree in Iran (Allahverdipour et al., 2021).

Pathogenicity of the representatives of *B. izbisi* strains was assessed by inoculating young walnut trees and immature fruits. Stem and shoot inoculations of walnut growing in pots demonstrated that all tested isolates were pathogenic on walnut. Although artificial inoculation by *B. izbisi* strains did not result in formation of oozing cankers, the development of extensive tissue necrosis and degradation of host tissues, as well as pathogen presence and re-isolation from tissues distant from the sites of inoculation, indicated that this bacterium is able to colonize walnut tissue, cause lesions, and survive for a long period in walnut trees. Similar difficulties in reproducing symptoms of external cankers on woody plants after inoculation with different *Brenneria* species including *B. nigrifluens, B. rubrifaciens, B. quercina*, and *B. rosae* subsp. *rosae*, were reported by other authors (Wilson et al., 1957; González et al., 2002; Biosca et al., 2003; Allahverdipour et al., 2021). Possible explanation for this could be the differences between natural infection conditions and artificial inoculations, the inoculation period, or due to physiological state of the tissues at the inoculation time and the following weeks (Biosca et al., 2003).

Although *B. izbisi* was the predominant species isolated from the symptomatic walnut trees, we also isolated strains of *Bn* and *Xaj* from the same walnut samples. The presence of more than one pathogenic species in the same plant samples, such as *Bn* and *Xaj*, was previously reported (Hajri et al., 2010; Allahverdipour et al., 2021). Moreover, Mazzaglia et al., detected the presence of both fungi and bacteria in walnut bark lesions that were associated with bark canker on English walnut (Mazzaglia et al., 2005). As it has been reported in some other pathosystems, such as acute oak decline (Denman et al., 2018) and olive knot disease (Buonaurio et al., 2015), specific symptom development might be a result of multispecies interaction. A recent study of Doonan et al. (2019) supports this idea and indicates the role of microbial communities and polymicrobial interactions in AOD establishment. Although we clearly demonstrated in this study that *B. izbisi* strains alone were able to cause tissue necrosis and lesions on inoculated walnut plants, other pathogens might possibly contribute to more severe disease development and have a synergistic effect. In addition, different environmental factors might contribute to disease development, as well as inadequate nutrient supply that may cause low tree vitality or to increase susceptibility for diseases. It has been demonstrated the relation between nutrient availability and the occurrence of watermark disease caused by *Bs* on *Salix alba*, showing that excess nitrogen makes willow trees fast-growing and more susceptible for watermark disease (De Vos et al., 2007). However, further studies are necessary to elucidate the role of each species and/or other factors in the deep canker symptoms development on walnut trees.

In order to facilitate rapid identification of the new walnut pathogen, we developed a specific PCR assay targeting the putative gene *hrpP* coding for type III secretion system HrpP C-terminal domain-containing protein. This assay enables identification of *B. izbisi* strains and their differentiation from other *Brenneria* spp., including closely related *Bs*, but particularly *Bn* and *Br* as causal agents of similar walnut canker disease.

This paper reports the description of a new bacterial species named *B. izbisi* associated with deep bark canker of the walnut in Serbia. This is an emerging disease of young walnut in Serbia that might have a high economic impact in the future, considering the increase of walnut production and its growing significance. The fact that the disease occurred only in young walnut orchards, indicates that pathogen might be introduced with planting material. However, the presence of the other widely distributed pathogens of walnut in the diseased trees, require further studies to clarify the role of each pathogen in the walnut bark canker development, their relationships and possible interaction with plant and other microbial communities.

### Description of *Brenneria izbisi* sp. nov.

*Brenneria izbisi* (iz.bi‘si. N.L. gen. n. *izbisi*, of IZBIS [Institut za zaštitu bilja i životnu sredinu, eng. Institute for Plant Protection and Environment], where this taxonomic study was performed).

Bacterial cells are Gram-negative and oxidase-negative, facultatively anaerobic, levan positive, nonfluorescent, and do not produce bright-red pigment on YDC and YPGA medium. The strains showed no pectinolytic activity on potato slices. They do not induce hypersensitive reaction on tobacco leaves but induced response on geranium plants. Positive for fermentation of glycerol, L-arabinose, D-ribose, D-galactose, D-glucose, D-fructose, D-mannose, L-rhamnose, inositol, D-manitol, methyl-α-D-glucopyranoside, N-acetylglucosamine, arbutine, esculine, salicin, D-melibiose, D-saccharose, D-rafinose, potassium gluconate and negative for erythritol, D-arabinose, D-xylose, L-xylose, D-adonitol, methyl-β-D-xylopiranoside, L-sorbose, dulcitol, D-sorbitol, methyl-α-D-mannopyranoside, amygdalin, D-celobiose, D-maltose, D-lactose, D-trehalose, inulin, D-melezitose, starch, glycogen, xylitol, gentiobiose, D-turanose, D-lyxose, D-tagatose, D-fucose, L-fucose, D-arabitol, L-arabitol, potassium 2-ketogluconate and potassium 5-ketogluconate (API 50 CHB/E). Nitrate is not reduced to nitrite, nor reduced to N_2_ gas. The strains do not produce indole, gelatinase, urease or acetoin but assimilate potassium gluconate and produce acid from glycerol.

The genomic G+C content of the type strain is 51.8 mol%. Its approximate genome size is 3.9 Mbp.

*B. izbisi* can be distinguished from other *Brenneria* spp. based on OGRIs (e.g. ANI and dDDH) and core-genome phylogeny, as well as by analysis of sequences of housekeeping genes. In addition, it showed unique phenotypic features, comparing with other *Brenneria* species.

The type strain, KBI 423^T^ (= CFBP 9035^T^ = LMG 32479^T^) was isolated from walnut trees in Serbia in 2020. Whole-genome shotgun sequence of the strain KBI 423^T^ has been deposited at the NIH GenBank under the accession number JAMPJU000000000.

## Data availability statement

The datasets presented in this study can be found in online repositories. The names of the repository/repositories and accession number(s) can be found in the article/**Supplementary Material**.

## Author contributions

KG and NK conceived and designed the study. KG and NZ performed the experiments, KG, NZ, and NK analyzed data and wrote the manuscript. All authors read, discussed, edited and approved the submitted version of the manuscript.

## Funding

The work of KG and NZ was supported by the Ministry of Education, Science and Technological Development, Republic of Serbia, Contract No. 451-03-68/2022-14/200010. The work of NK was funded by the Deutsche Forschungsgemeinschaft (DFG, German Research Foundation) – Project number 429677233.

## Acknowledgments

The authors would like to thank Prof. Aharon Oren (The Hebrew University of Jerusalem, Israel) for helpful advice on nomenclatural aspects. This research was enabled, in part, through computational resources provided by the BMBF-funded de.NBI Cloud within the German Network for Bioinformatics Infrastructure (de.NBI) (031A532B, 031A533A, 031A533B, 031A534A, 031A535A, 031A537A, 031A537B, 031A537C, 031A537D, 031A538A). We thank to Prof. Aleksa Obradović (University of Belgrade, Faculty of Agriculture) for constructive advices during research. In addition, we thank master student Marina Milošević for her contribution in rep-PCR and MLSA analysis.

## Conflict of interest

The authors declare that the research was conducted in the absence of any commercial or financial relationships that could be construed as a potential conflict of interest.

## Publisher’s note

All claims expressed in this article are solely those of the authors and do not necessarily represent those of their affiliated organizations, or those of the publisher, the editors and the reviewers. Any product that may be evaluated in this article, or claim that may be made by its manufacturer, is not guaranteed or endorsed by the publisher.

## Footnotes

1
https://www.fao.org/faostat/en/#data/QCL.

2
https://data.stat.gov.rs/Home/Result/13050301?languageCode=en-US.

3
http://iqtree.cibiv.univie.ac.at.

4
http://tree.bio.ed.ac.uk/software/figtree/.

5
https://github.com/widdowquinn/pyani.

6
http://ggdc.dsmz.de/distcalc2.php.

7
https://www.ncbi.nlm.nih.gov/pubmed/?term=22708584

8
https://lpsn.dsmz.de/genus/brenneria.

## Supplementary material

Supplementary Figure S1 Pathogenicity assay. Symptoms on walnut stem (A) two months after inoculation – no symptoms development on negative control (left), symptoms of tissue necrosis on the point of inoculation by the strain KBI 447. (B) 14 months after inoculation - no symptoms development on negative control (left), necrotic tissue under the bark extending deeper into the xylem (inoculation by the strain KBI 423) (middle and right). (C) Symptoms on young shoots three weeks after inoculation - no symptoms development on negative control (left), deep tissue necrosis around the point of inoculation (inoculation by the strain KBI 423).
