# Supplementary figures and images for: Polyphasic study of phytopathogenic bacterial strains associated with deep bark canker of walnut in Serbia revealed a new species, Brenneria izbisi sp. nov
Source: Front Plant Sci. 2022 Nov 24;13:1055186. doi: 10.3389/fpls.2022.1055186 (PMC9730526; doi:10.3389/fpls.2022.1055186)

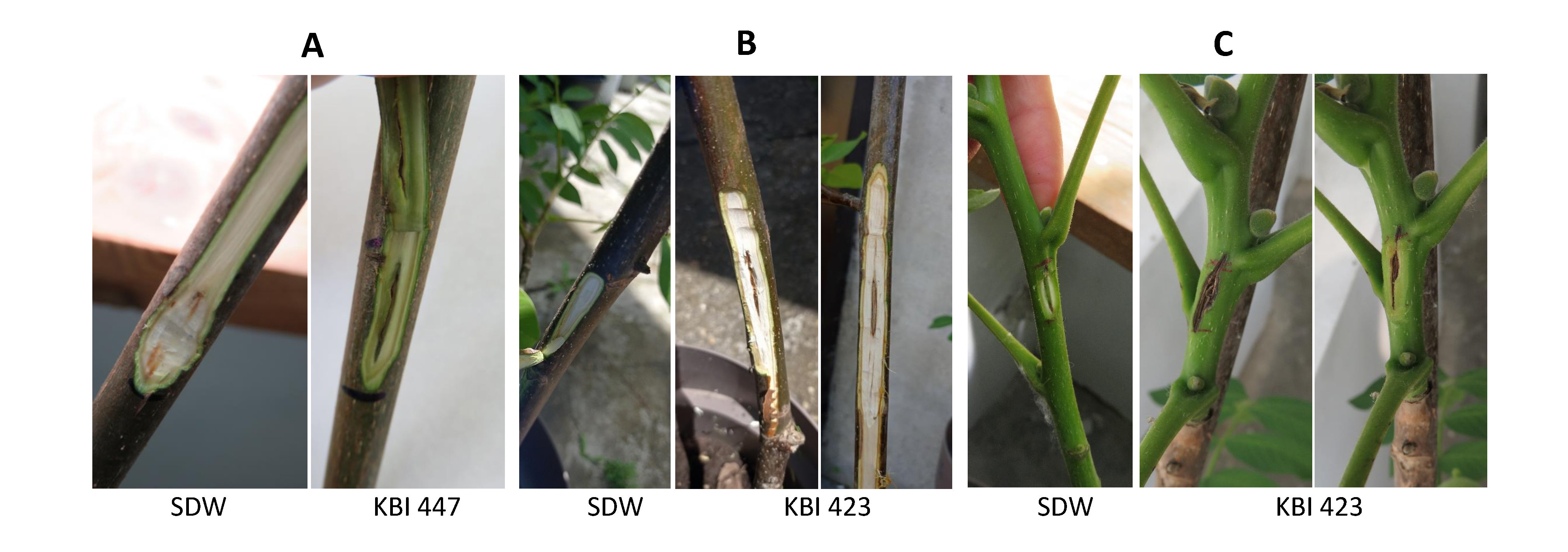

Supplement: Supplementary Figure S1 — Pathogenicity assay. Symptoms on walnut stem (A) two months after inoculation – no symptoms development on negative control (left), symptoms of tissue necrosis on the point of inoculation by the strain KBI 447. (B) 14 months after inoculation - no symptoms development on negative control (left), necrotic tissue under the bark extending deeper into the xylem (inoculation by the strain KBI 423) (middle and right). (C) Symptoms on young shoots three weeks after inoculation - no symptoms development on negative control (left), deep tissue necrosis around the point of inoculation (inoculation by the strain KBI 423). [file DataSheet_1.zip › Supplementary files/Figure S1.tif]
